# Supplementary material for: Are carbon emissions trading and green financial instruments synergistic? -Comprehensive quantitative research based on content analysis
Source: PLoS One. 2024 Mar 7;19(3):e0298601. doi: 10.1371/journal.pone.0298601 (PMC10919601; doi:10.1371/journal.pone.0298601)
Supplement: S1 Data — (ZIP) [file pone.0298601.s001.zip › Data/地级市Z_F工作报告.zip!/░▓╗╒/║╧╖╩╩╨/└┤╫╘╣1⁄2╓┌║┼í╛┬φ┐╦╩2╛▌═°í┐_2011.docx]

# **2011年政府工作报告**

发布时间：2011-01-12 10:02

各位代表：
                           
                           　　现在，我代表市人民政府，向大会作工作报告，请予审议，并请政协委员和其他列席人员提出意见。
                           
                           　　一、跨越赶超，“十一五”成就辉煌
                           
                           　　“十一五”是合肥科学发展、跨越赶超的重要时期。在省委、省政府和市委的坚强领导下，我们坚持以邓小平理论和“三个代表”重要思想为指导，深入贯彻落实科学发展观，抢抓国家促进中部崛起、扩大内需等战略机遇，积极应对全球性金融危机冲击，经受住复杂多变的宏观环境考验，有效化解了诸多矛盾，全面超额完成各项战略目标任务，使“十一五”成为合肥发展史上综合实力提升最快、城乡面貌变化最大、人民群众得实惠最多的时期。
                           
                           　　——过去五年，是经济持续快速发展，综合实力显著提升，实现争先进位的五年。
                           
                           　　全市地区生产总值实现翻番，年均增长17.5%以上，增速连续3年排名全国省会城市第一，总量由第18位前移至第15位。省会经济首位度由17.3%提升至22%。财政收入由130.9亿元增加至476.2亿元，增长2.6倍。全社会固定资产投资年均增长44%，累计完成9509.3亿元，是“十五”时期的6.7倍。城镇居民人均可支配收入和农民人均纯收入年均分别增长14.5%和16%以上，城乡收入差距比由3.02∶1缩小至2.8∶1。社会消费品零售总额由324.4亿元增加至839亿元，年均增长20.9%。城乡居民人均住房面积分别增长19.3%和14.5%。三次产业结构由5.7∶45.9∶48.4调整为5.0∶52.6∶42.4。金融、旅游、文化、会展、物流等现代服务业蓬勃发展，区域性金融、商贸物流、旅游会展和教育培训中心逐步形成。
                           
                           　　——过去五年，是县域经济实现大突破，新农村建设取得新成就，城乡统筹发展卓有成效的五年。
                           
                           　　坚持以城带乡、统筹发展，大力实施“县域突破”战略。累计投入涉农资金186.2亿元。县域经济在结构调整中迅猛发展。大宗农产品总产量连创历史新高，规模养殖比重上升到78%；农业增加值累计增长40%；农产品加工业产值突破500亿元。县域规模以上工业企业由171户增加至958户；工业增加值由45.1亿元增加至291.8亿元。县域生产总值由183.6亿元增加到630亿元，占全市比重由19.8%提高到23%以上。三县全部进入中部百强，肥西县跻身全国百强。
                           
                           　　新农村建设在深化改革中加快推进。开展农村产权交易、经营体制和城乡一体化试验区建设等改革试点。实施集体建设用地确权颁证和林权制度改革。以土地整理为抓手，深入开展整村推进、项目带动、产业培育等形式的新农村建设，万亩土地复耕和整村推进项目成为全国样板。建设市级以上示范村镇200个、农村集中区和新社区420万平米，34.5万农民居住条件得到明显改善。实施“村庄环境整治行动计划”、“清洁家园绿化乡村”等工程，创建3个国家级农业旅游示范点、8个省级环境优美乡镇、17个省级生态村。完成173座病险水库除险加固，解决74.3万人饮水安全问题。新建改建农村道路6200公里，所有行政村实现通水泥（油）路。城区道路、水电气热、公交等设施加速向县乡延伸。
                           
                           　　——过去五年，是新型工业化加速推进，自主创新能力显著提升，经济发展方式加快转变的五年。
                           
                           　　大力实施“工业立市”战略。坚持精力向工业集中、资源向工业汇聚、政策向工业倾斜，市本级累计投入专项资金118.7亿元。工业完成投资2826.4亿元，是“十五”时期的9.5倍、前十个五年计划总和的4.9倍。规模以上工业企业由668户增加至2091户，净增1423户；产值超亿元企业由102户增加至481户，超10亿元企业由19户增加至45户，超百亿元企业由1户增加至5户；规模以上工业增加值由288亿元增加至1052.7亿元，增长2.1倍。工业化率由36%提高至40%以上。支柱产业主导地位更加突出，家电业产值突破千亿元，成为全国最大的家电生产基地；汽车、装备制造业产值突破500亿元。战略性新兴产业加速培育，新型平板显示及光伏产业集聚发展，节能环保、生物、新材料、公共安全等产业迅速成长。
                           
                           　　以国家科技创新型试点市和合芜蚌自主创新综合试验区建设为抓手，大力实施“创新推动”战略。全社会科技研发投入占GDP比重由“十五”末的1.5%上升至2.3%。专利授权量年均增长45%，技术交易合同额年均增长28%。高新技术企业和创新型企业发展到710户，增加2.3倍；高新技术产业产值由488亿元增加至2000亿元。产学研模式取得突破，新建新能源汽车、语音信息、公共安全技术研究院等公共服务平台。企业创新能力显著提升，完成“双千工程”等技改投资1310亿元，新建国家级工程技术研究中心3个、企业技术中心9个，新增中国驰名商标8个、中国名牌产品6个。区域创新服务体系加快完善，示范核心区及“一中心、三基地”建设加速推进，省级以上科技企业孵化器发展到13个。人才资源对创新的支撑作用进一步显现，新建院士工作站6个，在肥工作院士达49人，17位海归人才进入“千人计划”。
                           
                           　　——过去五年，是现代化滨湖大城市框架迅速拉开，城市综合服务功能大幅提升，城市面貌发生巨变的五年。
                           
                           　　强力推进以路桥为重点的“大建设”，累计投入1074亿元；新建改造道路总里程783公里、桥梁99座，分别是“十五”时期的5倍和3倍。
                           
                           　　城市建成区面积由224.7平方公里扩大至310平方公里，市区常住人口净增近90万人，城镇化率由55.8%提升至65%。
                           
                           　　“141”空间发展战略成效明显。实施“大拆违”、老旧小区改造和景观整治，老城区功能不断完善、形象不断提升；城市副中心加快形成；滨湖新区快速启动，高水准规划、高起点建设，累计竣工建设面积640万平方米，建成道路81公里，路网围合面积30平方公里，初步形成可容纳30万人口的生态新城区。
                           
                           　　区域性综合交通枢纽框架初现。新桥国际机场建设进展顺利，航站楼主体工程基本完成；宁西铁路、沪汉蓉高铁相继建成，合蚌客运专线、合福高铁、铁路枢纽南环线及南客站加速建设，推动合肥迈向高铁时代。新建扩建合六、合淮阜等4条高速公路，绕城高速全线贯通，高速公路总里程增加一倍，形成“一环六射”路网格局。通江航道船闸提升工程加快建设，合肥港综合码头一期工程投入运营。
                           
                           　　市政基础设施建设大手笔推进。坚持强攻主动脉、健全微循环，强力推进畅通一环、打通中环、改造二环等工程建设。全面完成新一轮主城区小街巷改造。7条城市出入口道路改造基本完成，副中心“一刻钟快速交通网”初步形成。四大组团及开发区间实现畅通联结，主城区路网由“十字形”转为“方格网加放射状”格局。城市公用事业保障能力进一步提升，日新增供水能力44.5万吨，“川气入肥”工程成功实施，全省首座城市快速公交换乘枢纽及2条城市快速公交线路投入运营。
                           
                           　　——过去五年，是改革取得新突破，开放呈现新格局，发展活力持续增强的五年。
                           
                           　　超前谋划，深入推进重点领域和关键环节改革。市属国有中小企业和集体企业产权改革基本完成，合钢、美菱、荣事达等大企业改革重组成效显著。非公经济快速发展，新增私营企业及个体工商户4.5万家。新增上市企业7家，三县农村信用社改制全部完成，小额贷款公司试点达到39家。文化广电体制改革取得关键性进展，经营性文化事业单位转企改制基本完成。新一轮出租车经营权转换工作平稳。建立招投标新体制，成功交易1434.3亿元，资金节约和增值率25%。推行绩效预算、开门预算，完善非税征管方式，开展“小金库”专项治理，实行收支两条线管理，公共财政体制改革取得新突破。改革城市拆迁管理办法，实行“三榜公示”制度。探索建立规划、投资、建设等“六分开”的城市建设管理新体制及“借、用、还一体化”、“决策、管理、执行相分离”投融资管理新体制，得到中纪委及国家有关部委的充分肯定。建立土地管理委员会决策机制，打造“阳光地产”，成为全国首个节约集约用地试点市。
                           
                           　　坚持全方位开放，经济社会发展迸发巨大活力。加入长三角城市经济协调会，发起成立长三角会展城市联盟，与长三角地区实现一体化快速通关。合肥经济圈建设加快推进，与皖北地区开展结对合作。开通“港澳自由行”，增辟4条国际及地区航线，国际友城扩大到13个。整合招商资源，组建六批2600多个招商小组，累计招商引资4300多亿元，是“十五”时期的7倍多，新引进境外500强企业13家。对外贸易不断扩大，实现进出口总额近350亿美元。
                           
                           　　——过去五年，是环境综合治理加快推进，资源综合利用水平不断提升，城市生态明显改善的五年。
                           
                           　　水环境综合治理成效明显。投入46.1亿元，建成各类污水处理厂14座、污水管网1398公里，日污水处理能力从43.5万吨提高到96.2万吨，城市污水集中处理率达到85%以上；境内重点河湖综合治理成效显著，南淝河生态补水机制初步形成，巢湖西半湖氨氮、总氮较“十五”末分别下降49.6%、19.2%。
                           
                           　　城市空气质量保持良好。加快推广使用清洁能源，淘汰燃煤锅炉200多台（套），生活区集中供热面积达到1042万平方米；实施环保合格标志，强化机动车尾气排放管理，开通全国首条纯电动公交线路，99.5%出租车、29.4%公交车实现油改气。加强建设工地环境管理和“三车”整治，城市建设扬尘得到进一步控制。
                           
                           　　生态园林城市建设深入推进。围绕城市高速公路、铁路、高压走廊、重点河道等，建成一批生态长廊；坚持道路景观与公园建设并重，高标准实施徽州大道、紫云路等一批重点干道绿化，新建、扩建三国遗址、蜀峰湾等一批公园，基本形成“一圈、三环、四楔、五廊”的生态园林格局。城市建成区绿化覆盖率由37%提升至44.4%，人均公共绿地由8.7平方米增加到12.2平方米。
                           
                           　　资源节约型、环境友好型社会建设有效开展。经营性用地、工业用地平均容积率分别提高1和0.4，单位面积土地投资强度和实现税收均增加一倍以上。开展节水型社会建设和水生态系统保护与修复工作，农业灌溉水利用系数提高到0.49，万元GDP取水量降低18立方米。加快淘汰钢铁、水泥、小火电等落后产能，规模以上工业企业全部达标排放。万元GDP能耗下降21.7%，主要污染物排放量均低于省控指标。
                           
                           　　——过去五年，是民生显著改善，大局和谐稳定，社会建设全面发展的五年。
                           
                           　　坚持将改革和发展的成果更多地惠及人民群众，强力推进以改善民生为重点的社会建设。各级财政累计投入民生工程建设资金90多亿元，受益城乡居民500余万人次。
                           
                           　　就业和社会保障工作成就显著。积极建设国家创业型试点城市，不断完善积极的创业就业政策，实现由劳务输出向劳务输入的历史性转变，新增城镇就业岗位46.3万个，基本消除城市“零就业”家庭。成功举办两届“技工节”。开展新型农村养老保险试点，推进城镇居民医疗保险，解决未参保集体企业退休职工、被征地农民养老和大学生医疗及其他特殊群体保障问题。五项基本社会保险参保人次较“十五”末翻一番，实现城乡低保应保尽保和全面并轨。城区新建老年公寓36家。“515敬老工程”覆盖所有乡镇，荣获“中华慈善突出贡献奖”、“全国五保供养工作创新奖”。创建全国无障碍建设城市，残疾人社会保障和服务体系建设不断完善。加强城市低收入困难家庭住房保障，累计新建各类保障性住房264万平方米，完成城中村、棚户区改造538万平方米，发放廉租房补贴7055万元。
                           
                           　　教育事业全面发展。市本级教育经费投入实现翻番，由“十五”的24.4亿元增加到50.9亿元。新建中小学校87所，比前10年所建总和还多20所。学前教育方面被确定为国家改革试点市，义务教育均衡发展成为全国先进；外来务工子女定点学校由32所增加到115所，高中优质教育资源迅速扩大；合肥职教基地初具规模，民族教育成为全国样板，合肥学院应用型本科人才培养成效显著，民办教育呈集团化发展。大力实施布局调整和中小学标准化建设，全面完成D类危房改造任务。
                           
                           　　文化卫生体育旅游事业蓬勃发展。合肥大剧院、体育中心、广电中心、滨湖医院、市妇幼保健院东区等一批设施建成使用，渡江战役纪念馆、安徽名人馆、合肥美术馆加快建设。实施乡镇综合文化站、农村电影放映等惠农工程，建立全国首家“留守儿童图书馆”，创作出庐剧《村长娘子》等一批文化精品。广播电视综合覆盖率达100%。每千人口医院、卫生院床位达5.3张，累计投入乡村卫生基础设施建设资金6200余万元，新型农村合作医疗参合率98.9%，城市社区卫生服务网络覆盖率90%以上，城乡医疗救助体系不断完善。竞技体育争先进位，群众体育蓬勃发展。建设旅游精品工程，新增10家国家4A级景区、7家五星级酒店、2家全国百强旅行社。
                           
                           　　平安合肥建设及文明创建活动扎实推进。深入开展“大接访”、信访积案化解、矛盾纠纷排查化解等活动，圆满完成奥运圣火合肥传递等重大安保任务。和谐社区建设成果丰硕，蜀山、庐阳两区成为“全国和谐社区建设示范城区”。国防教育深入推进，连续荣获“全国双拥模范城”称号；民族团结进步工作获国务院表彰，宗教工作成绩显著。在全国首创人防、民防、政府应急“三办合一”模式，成为“全国人民防空先进城市”。强化社会治安防控体系建设，成为“全国社会治安综合治理优秀城市”，再获“长安杯”。广泛开展市容环境、取缔“正三轮”以及交通等专项整治，大力加强市民文明素质教育，成为“全国创建文明城市工作先进市”。安全生产监管扎实有效，各类事故逐年下降。消防、食品卫生、重大疾病防控等公共安全工作力度加大。侨务、地震、气象、统计、人口计生、档案、保密、妇女儿童、未成年人保护、地方志等事业取得新成就。
                           
                           　　——过去五年，是坚持依法治市，政府职能加快转变，行政效能大幅提升的五年。
                           
                           　　以规范行政行为和增强执行力为重点，深化行政管理体制改革创新，努力创造公开、公平、公正的制度环境。
                           
                           　　坚持依法行政。扎实推进“法治合肥”创建活动，圆满完成“五五”普法教育；规范行政处罚自由裁量权，在全国首创群众公议案件制度，促进行政权力“阳光运行”。自觉接受人大监督、政协民主监督和社会监督，累计办理人大代表议案39件、建议759件，政协提案2151件；提请市人大常委会审议通过地方性法规12件，修订、废止法规18件；修改、废止政府规章50件；修订政府规范性文件29件，废止245件，宣布失效405件。坚持科学民主决策。健全以《市政府工作规则》为总纲的政府工作制度；建立市政府常务会学法及人大代表列席制度，完善政府法律顾问、重大决策专家咨询等制度。推进政务公开、政府信息公开，加快电子政务建设，整合“网上信箱”、开办政民直通车，持续开展政风行风评议活动，解决了一批热点、难点问题。坚持廉洁从政。严格落实《廉政准则》和党风廉政建设责任制，规范领导干部从政行为。健全和完善土地使用权和国有资产出让、政府采购等相关制度247项；加强审计监督，落实“双百”制度，强化中央财政投资和国债资金项目监管，开展土地管理、市场价格调控等专项督查，严肃查处各类违法违纪案件。提升政府效能。完成市县政府机构改革，在全国率先推出公务员转任立法、开门抓“三定”等创新举措。将原来的多种服务热线整合为统一的“12345”政府服务热线。对行政审批项目进行5轮清理，减少行政许可事项390项，下放审批权限120多项；实行“缺席默认”、“全程代办”、“并联审批”等具有合肥特色的改革措施。加强作风建设。深入开展学习实践科学发展观、“查摆找补创”、“创先争优”等活动，机关服务意识进一步增强。大力推进节约型政府建设，着力降低行政成本，市直行政事业单位公用经费、出国、公务接待、车辆购置费等连续五年“零增长”。
                           
                           　　各位代表！刚刚过去的2010年，是“十一五”发展的收官之年，我们坚持把保持经济平稳较快发展作为首要任务，加快经济发展方式转变和经济结构调整，强力推进自主创新，深入实施各项改革，突出保障和改善民生，着力维护社会和谐稳定，较好地完成了市十四届人大三次会议确定的各项任务。初步统计，全市实现地区生产总值2600亿元以上，同比增长17%以上；财政收入476.2亿元，增长39.3%，其中，地方财政收入259.4亿元，增长43.4%；全社会固定资产投资3067亿元，增长24.2%；社会消费品零售总额839亿元，增长19.8%；居民消费价格指数涨幅控制在2.7%；城镇居民人均可支配收入19051元，增长11%；农民人均纯收入超过6800元，增长12%以上；城镇登记失业率控制在4.3%以内。
                           
                           　　2010年的主要工作：
                           
                           　　一是强力推进重点产业项目建设。抢抓政策叠加机遇，全力谋划推进产业层次高、带动能力强的大项目、好项目。工业经济亮点纷呈，长安汽车合肥基地、友达光电、赛维LDK等一批产值超百亿元项目开工建设，京东方六代线、鑫昊等离子、彩虹高世代液晶玻璃基板、熔安动力等竣工投产，彩虹蓝光LED、中盐化工基地等项目加快建设。自主创新深入推进，世界首个城域量子通信试验示范网开工建设。现代服务业加速发展，国际金融后台服务基地建设取得突破，工总行、建总行、浦发银行后台中心项目相继动工；省级商贸物流开发区获批筹建，万达广场等一批高端服务业项目建成开业。
                           
                           　　二是高度重视和加强“三农”工作。全面落实各项强农惠农政策，发放各类涉农补贴9.8亿元。县域经济加快发展。粮油生产连续第七年增产丰收；规模农业面积突破150万亩；组织实施农业“百园示范工程”，新增农业园区23个、规模养殖场320个、市级以上龙头企业62家，肥东和长丰农产品加工园区成为首批省级农业产业化示范区。农村社会化服务水平进一步提升，新增农民专业合作组织207个，培育新型农民2.4万人。农村基础设施建设进一步改善。完成7座中型水库、14座小（1）型、28座小（2）型病险水库改造；提级改造农村公路360公里，三县行政村通班车率达到99%以上；新建饮水安全工程21处，解决17.5万农民饮水安全问题。农村社会保障水平进一步提高。46.3万人参加新农保试点，13.6万名农村60周岁以上老人领取60元/月基础养老金；三县城镇居民医保和新农合全面并轨，在全省率先实现城乡居民医保一体化县域全覆盖。完成农村造林5.2万亩，启动3个土地整治和整村推进项目。
                           
                           　　三是继续深化改革开放。加大国有企业“集中主业、退出辅业”及同类资源优化整合力度，涉改企业生活区移交工作进展顺利。皖通科技、荃银高科发行上市，高科创业等风险投资机构加速引进。创新基层医疗卫生机构人事及收入分配制度，药品零差率销售有效执行，城乡居民就诊费用明显下降。土地管理制度改革进一步深化，节约集约用地成效显著。整合新站、瑶海及肥东、长丰部分区域规划与管理体制，规划建设合肥承接产业转移示范区。合肥出口加工区获批设立。成功承办徽商大会、自主创新要素对接会、家电博览会、中国企业500强发布会，隆重举行与全国知名民营企业合作对接、与久留米市结好30周年庆典等活动。
                           
                           　　四是快速度推进城市基础设施大建设。继续完善各项建设规划，全力推进区域综合交通枢纽及市政基础设施建设。全年新建续建工程516项，建成道路73公里，完成桥梁12座，建成水环境工程13项。合肥火车站改造工程完工，机场高速、合店路二期开工建设，长江西路高架及一批支路网建成通车，南北高架一号线、裕溪路高架及轨道交通一号线试验段建设加快推进。城市生态不断改善。小仓房污水处理厂建成试运行，经开区污水处理厂二期工程开工建设；排水设施百日会战成效显著，梳理整改问题1700多个；再掀绿化造林新高潮，新增城市园林绿地1.1万亩；开展生活垃圾收运体系改革，全面完成省节能减排目标任务。三大开发区及滨湖新区开通支线公交。
                           
                           　　五是统筹发展社会事业。12所市属义务教育学校移交辖区管理。超额完成校舍安全工程年度目标任务，加固、重建面积达220多万平方米；全面完善校园安保工作。举办第六届中国曲艺牡丹奖合肥赛区比赛等文化演出活动400余场次。成功承办第四届全国体育大会，实现了“精彩圆满、富有特色、令人难忘”的办会目标。参加省十二运会，获金牌、奖牌、团体总分三项第一。积极推进基层社区建设和管理，被列为全国社会管理创新综合试点城市。圆满完成第六次全国人口普查入户登记工作。
                           
                           　　六是大力保障和改善民生。实施33项民生工程，各级财政投入24亿元，增长16%。全面落实企业职工基本养老保险省级统筹，对未参保大集体企业退休人员实施补保政策。继续完善农村低保制度，保障困难群众10万多人。完成残疾人专项普查，对2.4万名贫困重度残疾人实施救助。在全省率先取消城乡低保、农村五保户、重点优抚对象住院门槛费。城市居民最低生活保障标准由260元提高到280元。新建续建保障性住房和复建点工程67项，总投资117.1亿元，其中，廉租房项目21个、竣工6090套，公租房开工面积超过100万平方米，完成城中村及棚户区改造14693户。加强“菜篮子”等居民基本生活必需品价格调控监管，建立快速反应机制，扩大供货渠道，切实保障市场供应和困难群众正常生活。全力做好信访工作，强力整治社会治安突出问题，狠抓安全生产隐患排查治理，深入开展“三车”交通安全专项整治。
                           
                           　　各位代表，回首“十一五”，我们坚持科学发展，不断深化对经济社会发展规律的认识，在实践中探索了一条具有合肥特色的加速崛起之路，谱写了合肥发展史上的绚烂篇章，经验弥足珍贵。
                           
                           　　这一时期坚定的发展思路是，坚持发展第一要务、招商第一要事，大力实施工业立市、县域突破、创新推动、“141”空间布局等重大发展战略，凝心聚力，从容应对复杂形势和挑战，坚定不移地加快新型工业化、城镇化和农业现代化。
                           
                           　　这一时期强劲的发展动力是，坚持解放思想、深化改革、扩大开放，不断打破陈旧和僵化的思想桎梏，着力释放全社会发展活力，成功探索并建立了一套有利于加快发展、被誉为“合肥模式”的体制和机制。
                           
                           　　这一时期根本的发展方法是，坚持统筹兼顾，协调推进，在全面提升城市二、三产业发展水平的同时，大力支持县域经济发展，城乡统筹发展力度越来越大。在加快经济发展的同时，高度重视社会建设，大力实施民生工程，重点解决好社会关心的入学就医、劳动就业、社会保障等热点难点问题。
                           
                           　　这一时期浓厚的发展氛围是，坚持正确的政绩观，谋划工作重长远、推动工作重细节、落实工作重成效，确保兑现对群众的承诺；大力推进效能革命，发展环境不断优化，政府的执行力、公信力和行政效率大为提升。
                           
                           　　各位代表，五年的发展历程波澜壮阔，五年的成就让我们倍感自豪。这是省委、省政府和市委正确领导的结果，是市人大依法监督、市政协民主监督、社会各界大力支持的结果，是全市人民锐意改革、顽强拼搏的结果。在此，我代表市人民政府，向辛勤奋战在各个领域、各个岗位，为合肥发展作出贡献的全市人民，向驻肥人民解放军、武警官兵、公安干警和中央驻肥单位，向各民主党派、工商联、无党派人士、各人民团体和社会各界人士，向所有关心支持合肥改革开放与现代化建设的海内外朋友，表示衷心的感谢和崇高的敬意！
                           
                           　　在肯定成绩的同时，我们也清醒地看到存在的问题和不足：经济总量还不够大，经济结构调整和发展方式转变任务还很重；经济发展与社会事业之间、城乡之间发展还不平衡；社区特别是农村社区基本公共服务水平还较低，社会安全稳定的基础仍较薄弱；机关作风建设还需不断改进，依法办事意识还需进一步增强，等等。这些都是我们下一步工作中亟待改进的。
                           
                           　　二、“十二五”发展的指导思想、主要目标和重点任务
                           
                           　　“十二五”是合肥经济社会进一步加快发展、转型提升的重要时期。随着国内外产业资本加速转移，国家促进中部崛起战略、扩大内需战略及皖江城市带承接产业转移示范区建设深入实施，合肥发展的比较优势更加凸显；工业化、城镇化加速推进，产业和消费加快升级，持续投入效应不断释放，合肥发展的内生动力显著增强。我们完全有条件推动合肥经济社会在新的起点上实现更长时间、更高水平、更好质量的发展。我们要以永不自满、永不松懈的劲头，瞄准更高的定位，勇攀发展新高峰。
                           
                           　　“十二五”经济社会发展总的指导思想是：以邓小平理论和“三个代表”重要思想为指导，深入贯彻落实科学发展观，落实省委、省政府对合肥发展的新要求，以科学发展为主题，以加快转变经济发展方式为主线，以富民强市为目的，着力推动科学发展、跨越发展、和谐发展，加快建设现代化滨湖大城市和现代产业基地，努力朝着区域性特大城市目标迈进，进一步增强合肥在全省发展大格局中的龙头带动作用。
                           
                           　　今后五年的主要奋斗目标是：地区生产总值年均增长16%，规模以上工业增加值年均增长20%，财政收入年均增长16%，城乡居民收入在“十一五”末基础上翻一番；城市建成区面积扩大到410平方公里，城镇化率75%；全面完成节能减排各项任务。
                           
                           　　实现“十二五”发展目标，重点要把握和推进五个方面工作：
                           
                           　　（一）坚持科学发展，加快推进经济发展方式转变。
                           
                           　　强化在发展中促转型、在转型中谋发展，持续扩大有效投入，着力优化投资结构，加快构建现代产业体系。大力实施“1346”行动计划，加快推进一批带动力强的重大项目建设，增强经济发展的内生动力。强力推进国家创新型试点城市、合芜蚌自主创新综合试验区建设，以创新为依托推动新型工业化发展，做大做强优势产业、改造提升传统产业、大力培育战略性新兴产业，打造新型平板显示、新能源、家用电器3个两千亿元级产业和汽车及零部件、装备制造、食品及农产品加工3个千亿元级产业，培育2～3家产值超500亿元和30家产值超百亿元的企业集团。坚持把发展规模高效农业作为建设现代农业第一工程，着力构建“种、养、加”一体化的现代农业产业体系。推动生产性服务业集聚化发展、生活性服务业便利化发展，加快形成区域性金融物流、文化会展、商贸旅游、要素资源交易、服务外包等现代服务业中心。
                           
                           　　（二）坚持城乡一体，加速构建区域性特大城市基础架构。
                           
                           　　坚持“主城提质、组团扩容、打造新市镇、建设新农村”，加大城乡统筹发展力度，深入实施“141”空间发展战略。以提升城市功能品位，增强综合承载力为导向，规划建设一批战略性、枢纽型、保障性基础设施项目。加快机场、铁路、高速公路、港口和城际轨道交通等立体交通体系建设，基本建成全国重要的综合交通枢纽。坚持打通主动脉、完善微循环，继续推进“141”组团快速路网体系和市域内干支路网系统建设。按照“适度超前、持续发展、保障有力”的要求，加快水电气热等要素支撑体系建设。加强农村道路、水利等设施建设，进一步完善合肥城市防洪体系，完成中小水库除险加固任务。优化水资源配置，加强饮用水水源地保护。深入实施“千村百镇”等新农村建设示范工程，改善村容村貌，建设农民幸福生活的美好家园。
                           
                           　　（三）坚持加快生态建设，推动经济社会可持续发展。
                           
                           　　按照“宜居宜业、独具魅力”的要求，打造水清、天蓝、树绿、气爽的生态宜居城市。完善城市功能区划，推进产业合理布局。大力发展循环经济，加快节能减排技术推广应用。严格执行国家产业准入标准，加快淘汰落后产能。推进“引江济巢”工程建设，加强巢湖水环境综合治理，提升城市污水集中处理率，力争实现全收集、全处理，进一步做好“水文章”。加大空气、噪音等污染防治力度，推进生活垃圾收运体系改革，提高污泥、固体废弃物和农作物秸秆等综合利用水平。继续实施农村环境综合整治，控制和减少面源污染，建设“清洁家园”。大力推进生态文明建设，持续做好“绿文章”，森林覆盖率超过全国平均水平，争创国家生态园林城市和森林城市。
                           
                           　　（四）坚持深化改革扩大开放，持续增强发展活力和动力。
                           
                           　　解放思想，敢为人先，进一步消除制约经济社会发展的深层次体制机制障碍，加快形成活力开放的投资发展环境。以土地管理、户籍制度、农村金融等为重点，深入推进城乡一体化综合配套改革试验区建设，引导优势资源向农村集聚。稳步推进行政管理体制改革，转变政府职能，提高行政效能。分类推进文教卫、城市管理等事业单位改革，不断深化国资监管体制改革。促进土地、技术和劳动力等要素市场发展。进一步完善财权与事权相匹配的财政管理体制，形成有利于可持续发展的地方金融服务体系。开展大招商，主动承接产业转移，大力引进投资规模大、产出效益好、带动能力强的大项目。加强与国内外友好城市、产业转移重点地区、合肥经济圈城市、省内结对发展市县的交流与合作。
                           
                           　　（五）坚持加强社会建设和管理，着力提高人民生活水平。
                           
                           　　坚持群众利益至上，大力实施民生工程，加快发展社会事业，让人民群众生活得更加幸福、更有尊严。建立统筹城乡的基本公共服务体系，推动全民创业，不断完善就业、基本养老、最低生活保障和社会救助制度，推进城镇居民基本医疗保险、城镇职工基本医疗保险和新型农村合作医疗制度建设，形成覆盖城乡的社会就业、救助体系和现代养老服务体系。推进保障性住房建设，加快城中村及棚户区改造，切实保障人民群众基本住房需求。以创建全国文明城市为统揽，全面提高城市品位和市民综合素质。坚持教育优先发展，推进教育体制改革，提高教育质量。坚持文化强市、人才兴市，加大公共文化、体育等基础设施建设，发展新闻出版、广播影视、文学艺术等事业；建立文化市场综合管理体系。突出大服务思想，打造大综治格局，提高应对、处置公共突发事件能力，全面推进全国社会管理创新综合试点市建设。
                           
                           　　三、团结奋进，全力做好2011年各项工作
                           
                           　　2011年是“十二五”规划的起步之年，也是建党90周年，做好今年的各项工作，意义重大。综合考虑，今年经济社会发展的主要目标是：地区生产总值增长16%，财政收入增长15%，全社会固定资产投资增长22%，社会消费品零售总额增长19%，城镇居民人均可支配收入增长13%，农民人均纯收入增长14%，城镇登记失业率控制在4.5%以内，人口自然增长率控制在7‰以内，居民消费价格指数涨幅控制在4%左右，单位GDP能耗下降率及主要污染物减排量达到省控目标。
                           
                           　　实现上述目标，着重要做好以下九个方面工作：
                           
                           　　（一）继续扩大有效投入，进一步增强加快发展的后劲。
                           
                           　　围绕支柱产业发展、战略性新兴产业培育和重大基础设施建设等，加快谋划、储备一批符合国家产业政策导向，管全局、利长远、带动性强的重大项目，不断形成持续的投资后劲。按照抓开工、促在建、保投产的要求，强化调度，加快重大项目建设，确保列入开工计划的项目尽快落地、列入竣工计划的项目尽快建成投产。继续实施“1346”行动计划，大力推进京东方8.5代线、赛维LDK、长安汽车合肥基地、友达光电、合肥电厂6#机组、中盐化工基地等一批重大项目建设。
                           
                           　　进一步拓宽融资渠道。继续做好银企对接服务，积极争取扩大信贷投放。扩大直接融资规模，力争设立1～2支产业基金，引进或新设2～3支股权投资基金，5～6家企业上市首发。围绕产业链、企业集群，狠抓6个千亿元级重点产业招商，大力引进超50亿元、百亿元的大项目。建立重大招商活动签约项目信息库，推动签约项目早注册、早开工、早投产。完善投资促进政策，支持非公经济发展，激活民间投资，加快形成市场主导的投资内生增长机制。
                           
                           　　（二）深入推进“工业立市”战略，促进经济持续较快增长。
                           
                           　　坚定实施“工业立市”战略，调整结构，做大总量，提升产业竞争力，保持工业在经济发展中的龙头带动作用。
                           
                           　　加强工业运行调节，密切关注重点产品、原材料价格和市场需求动态，保障煤、电、油、运等生产要素供给。探索建立企业帮扶工作长效机制，加快高成长性企业培育步伐。实施“双千工程”，支持汽车、家用电器、装备制造等支柱产业加快发展和冶金、建材等传统产业改造升级，力争技改投入占全部工业投资50%以上。突出加强工业招商引资工作，深化与中央企业及国内知名民营企业的合作对接，吸引国内外有实力企业来合肥发展，推动新型平板显示、太阳能光伏、新能源汽车、公共安全等战略性新兴产业集聚发展。加快国家家电质检中心建设，深入开展质量兴市活动。大力实施技术标准、商标品牌战略，帮助企业抓好产销衔接和市场开拓。强化开发园区基础设施建设，提升产业承载和配套服务能力。
                           
                           　　加强工业、商贸、交通等重点领域节能改造，严格电力、钢铁、化工、建材等重点行业节能管理；加快推广节能新技术、新产品，开展节能汽车、节能电机、节能空调等新产品能效检测，建立一批节能新产品使用企业示范点。
                           
                           　　（三）加快构建区域创新体系，努力提升自主创新能力。
                           
                           　　深入推进国家创新型试点城市和合芜蚌自主创新综合试验区建设，努力在科技、产业、人才和改革成果上取得新突破。实现高新技术产业产值2500亿元，全社会科技研发投入增长25%以上。
                           
                           　　加快推进创新服务平台建设。发挥“一中心、三基地”带动功能，引进和建设一批国家级科技创新载体、共性技术和关键技术创新平台，完善新能源汽车、语音信息及公共安全等产业研究运行机制。
                           
                           　　不断提升企业创新能力。引导产业龙头企业与高校、科研院所合作建立产业技术创新战略联盟。围绕6个千亿元级产业和战略性新兴产业，优选10家左右企业创建“品牌示范企业”。支持企业研发平台建设，新建工程、企业技术中心100家。继续实施创新型企业培育工程，新增高新技术和创新型企业120家。实施“雏鹰企业培育计划”，重点扶持100家具有爆发式增长潜力的中小企业。
                           
                           　　进一步优化创新发展环境。争取进入国家科技金融创新试点，突出抓好政策性担保、科技贷款风险补偿、“新三板”市场试点等工作。扩大知识产权、股权质押贷款规模，鼓励优质科技型中小企业发行集合债券。支持开展创新人才激励试点、企业股权和分红激励改革，大力吸引海外高层次人才、归国留学人员及团队来肥创新创业。
                           
                           　　（四）统筹城乡一体化发展，推动新农村建设取得新突破。
                           
                           　　进一步发挥中心城市辐射带动作用，统筹城乡规划，统筹城乡空间布局，统筹城乡建设，促进资源优化配置，加快推进城乡统筹一体化发展。
                           
                           　　进一步加快农业和农村产业结构调整。大力发展现代高效规模农业，深入实施种植结构调整、畜牧提升、水产跨越和农业产业化“双百双千”工程，推进农业园区和农业产业化示范园区建设，加速农业转型升级。新增特色高效农业面积5万亩、规模养殖场（区）100个、水产养殖面积2万亩，形成一批年产值超10亿元的高效农业产业集群。
                           
                           　　不断加快新农村建设步伐。完善市县融合发展机制，强化市域城镇体系规划，重点推进城市副中心和建制镇建设，提升县域发展水平和综合承载力。完成在建万亩土地整理项目区耕地复垦整理，完善项目推动、产业培育、土地整治和整村推进建设模式。进一步健全农村公共服务体系，推进新农村示范片区建设，高起点建设一批新型农村社区。
                           
                           　　推动创业、就业促进农民增收。规范开展农民工技能培训、“阳光工程”培训和新型农民科技创业培训，深入实施农民转移工程，力争全年新增5万农民转移就业。加强农机、农技等农村社会化服务体系建设，完善农产品质量安全监管，提高农业综合执法能力。
                           
                           　　推进农村基础设施建设。加强重点水利工程和农田水利配套设施建设。实施农村公路提级延伸连网工程，加快农村安全饮用水工程建设进度。大力推广沼气、太阳能等能源利用。完成广播电视“村村通”工程，巩固提高农村电网改造成果。
                           
                           　　（五）继续推进基础设施建设，不断增强城市综合承载力。
                           
                           　　按照区域性特大城市和构建合肥经济圈的要求，编制新一轮合肥近期重大基础设施建设规划，推进土地利用总体规划报批。
                           
                           　　全面建设全国重要的综合交通枢纽。基本建成新桥国际机场各项设施。大力推进铁路枢纽及南环线、合福线、合蚌客运专线及合肥西站工程建设，开工建设西合复线。全力推进合宁城际铁路前期工作。加快实施合安高速小西冲至紫云路“四改八”工程、巢湖船闸及裕溪船闸工程，开工派河港区、合裕航道疏浚等项目，做好丰乐河、店埠河航道整治前期工作。继续大力推进出城口道路建设，确保新蚌埠路二期、南淝河大桥、合店路二期完工通车，推进环巢湖道路、合白路、合马路改造工程，新建莲花路跨派河大桥。加快推进南淝河防洪工程建设。
                           
                           　　继续推进城市道路基础设施建设。加快轨道交通一号线建设，适时启动轨道交通二号线，实施合作化路、铜陵路、阜阳北路、包河大道高架及胜利路畅通工程，建成裕溪高架及南北高架一号线。开工建设淮海大道、临泉东路、方兴大道等骨干道路，同步实施下塘路、皖河路等支路网工程。
                           
                           　　不断完善中心城区功能。加快推进老城区改造和功能品位提升，全面完善排水设施雨污分流和水电气热配套工程。滨湖新区重点围绕建设生态、文化之城的目标，加快塘西河“两坝三闸”工程和金斗公园、塘西河公园、方兴湖公园建设；加快巢湖岸线生态整治及蓝藻防控工程，推进堤防加固、防护林种植和滨水景观建设；加快滨湖国际会展中心、合肥博物馆和要素大市场等项目建设，启动城市规划展览馆建设。加快政务文化新区重点商业配套设施建设，完善中心商务区功能。
                           
                           　　继续加强城市生态环境建设。实施十五里河、二十埠河河道治理、经开区污水处理厂二期工程，开工建设陶冲污水处理厂。改革生活垃圾收集方式，提升生活垃圾收运密闭化及处理无害化、减量化水平；积极推进垃圾焚烧发电、龙泉山垃圾处理厂二期、污泥及固体废弃物焚烧处置热源综合利用等工程建设。围绕创建国家生态园林城市和森林城市，重点实施一批森林生态廊道和城市生态公园工程。建设天水公园、菱湖公园、繁华公园、陶冲湖公园一期，提升改造大蜀山森林公园，启动合肥植物园扩建工程。实施南淝河上游、两大水库、巢湖边岸成片造林，高标准提升南外环高速、合安高速等绿色长廊，推进村庄绿化、乡村道路、农田林网和渠道塘坝等造林绿化。
                           
                           　　（六）围绕重点领域和关键环节，进一步深化改革。
                           
                           　　全力推进新一轮国有企业改革。完成合钢集团整体破产，依法实施国风、荣事达、物资集团改革注销工作，推进国有资本股权对外合作发展。深化公用事业企业“集中主业、退出辅业”改革，全面完成企业生活区移交工作。
                           
                           　　统筹推进行政体制和事业单位改革。科学界定市、区财权和事权，加快城市管理体制改革和管理重心下移。完善市对区财政管理体制，促进开发区与县区工业园区合作发展。完善招投标监管体系，推进市场要素资源进场交易。加快基层医疗卫生体制改革，全面执行国家基本药物制度，完善公办医疗机构用人机制和绩效工资考核制度。深化公益性文化事业单位人事、收入分配、社会保障改革，完成文化市场综合执法改革、国有艺术院团改革，组建文广演艺集团公司。继续深化土地管理制度改革，强力推进国家节约集约用地试点市建设，完善土地利用双向约束机制，落实项目投资强度和税收目标，提高土地利用效益。
                           
                           　　（七）大力发展现代服务业，加快提高城市综合竞争力。
                           
                           　　围绕建设区域性金融中心、商贸物流中心和旅游会展中心，加快现代服务业提档升级，促进现代服务业与新型工业化融合发展。
                           
                           　　继续加快发展生产性服务业。积极引进股份制商业银行、外资银行、优质异地城市商业银行以及各类保险机构来肥设立区域性总部、后台服务中心或分支机构。稳步推进股份制商业银行在县域布点。积极筹建金融租赁公司，以开明开放的举措推进地方金融体系建设。推动航空、新港、铁路货运枢纽物流园区及城际公路货运站场等重大项目建设，促进中国合肥（国际）农产品物流园和撮镇商贸物流开发区建设，争取引进若干国内外一流物流企业，不断提升物流业专业化、现代化水平。加大对专业展会的扶持力度，办好家电博览会、自主创新要素对接会等品牌会展活动，力争全年展会总量达160场。
                           
                           　　进一步提升现代生活性服务业水平。继续加快推进新老城区中央商务区及一批特色商业街区建设改造，引进高端百货和知名连锁超市，改善购物环境，提升商业规模和档次。深入推进“万村千乡”和“新网”工程，积极推进标准化菜场、“农超对接”及再生资源回收体系建设；继续实施“早餐工程”、“双进工程”，加快社区商业服务设施建设。进一步做好家电下乡、汽车及家电“以旧换新”等工作。加强市场和价格监管，建立粮食等重要大宗商品储备。深化合肥经济圈“5+1”文化旅游合作，促进文化场馆与旅游项目融合。培育旅游航线、引进航空基地公司，打造一批旅游综合体，建设国际滨湖旅游目的地。优化供应结构，推进住宅产业化和节能省地环保型住宅建设，引导房地产业稳定健康发展，提升物业管理服务水平。
                           
                           　　大力发展服务外包产业和其他新兴服务业态。推进服务外包示范城市建设，以软件研发、呼叫中心、动漫网游、工业设计等为重点，实施新注册大型企业一次性奖励、领军人才资助等政策，大力引进全球100强、国内50强企业。
                           
                           　　（八）协调发展各项社会事业，大力保障和改善民生。
                           
                           　　进一步提标扩面，加大民生工程投入，努力推进基本公共服务均等化。
                           
                           　　坚持教育优先发展。全面启动国家级学前教育改革试点工作。继续推进义务教育均衡发展、学校标准化建设、中小学布局调整，新建和改扩建168中学东校区、和平中学等9所中小学，鼓励支持民办教育事业发展。完成校舍安全工程年度规划建设任务。重视发展老年教育，关注留守儿童身心健康。加快合肥职教基地建设步伐，完善配套设施，推进合肥工业学校、经贸旅游学校等建设。支持民办院校、合肥幼儿师范学校升格及合肥学院争创高水平应用型本科高校。
                           
                           　　大力推进医疗卫生文化体育事业发展。巩固和完善新型农村合作医疗制度，全面推进城镇居民医保和新农合统筹并轨。健全基层医疗卫生机构的补偿和运行机制，推进公共卫生机构规范化建设。加快乡镇卫生院、村卫生室建设，实现达标率100%；以能力建设为重点，提升城市社区卫生服务水平。加强公共卫生体系建设和食品药品安全监管，大力开展爱国卫生运动和健康城市建设。继续推进乡镇综合文化站、电影“2131”工程建设，精心筹办第二十届金鸡百花电影节、第十一届中国国际合唱节。全力备战第六届全国城运会，推动全民健身运动深入开展。积极建设全国婚育新风进万家活动示范市，开展全国流动人口计划生育基本公共服务均等化试点。积极发挥工会、共青团、妇联等人民团体作用，做好民族宗教及人防、消防、气象、地震、外事侨务、统计、档案、保密等工作。制定实施新一轮《合肥市妇女儿童发展纲要》。完成二轮《合肥市志》编纂任务。
                           
                           　　继续推动就业和社会保障工作。大力推进国家级创业型试点城市建设。整合和规范人力资源市场，建设大学生就业创业见习基地，鼓励和引导大学生到基层灵活就业和创业，开展跨地域人力资源协作与交流。继续实施“零就业家庭”、“4050”人员、残疾人等困难群体就业援助工作。认真做好被征地农民社会保障、新型农村农民养老保险工作，完善企业职工基本养老保险制度，扩大工伤保险覆盖面。探索建立城镇居民养老保险制度，推动社会保障管理服务社会化，逐步实现各项社会保险关系无障碍转移接续和医疗保险异地结算。深入开展第三批标准化示范社区建设，力争三县25%的建制村转变为社区。加大医疗救助力度，积极探索“支出性贫困”家庭救助工作新模式，建立市、县区城乡医疗救助资金筹资标准及城乡低保标准自然增长机制。实施贫困重度残疾人生活救助、贫困残疾人康复救助等工程，推进慈善事业和城乡一体的社会救助及残疾人社会保障体系建设。鼓励发展各类养老机构，完成“社会福利中心”一期工程建设；开展居家养老服务社会化试点，建设18个街道养老服务中心、140个社区养老服务站。大力推进保障性住房建设，加大公租房及廉租房建设力度，加快城中村及棚户区改造工作。做好优抚安置工作，争创全国双拥模范城“七连冠”。
                           
                           　　着力维护社会稳定。以项目为抓手，推进全国社会管理创新综合试点市建设。加快推进智能交通、综合地理人口信息系统建设，加强城乡社区警务、群防群治等基层基础建设，突出抓好重点地区社会治安综合治理。大力加强民兵预备役等国防后备力量建设和人防民防应急工作。深化隐患排查治理和重点行业、领域集中整治，深入推进安全生产达标创建活动。积极探索建立社会矛盾源头预防化解机制，深入开展领导干部接访和下行联合接访工作。持续深入开展“法治合肥”创建活动，启动实施“六五”普法。
                           
                           　　（九）切实加强政府自身建设，不断提升服务能力和水平。
                           
                           　　抢抓发展机遇，应对发展挑战，实现发展目标，必须切实加强政府自身建设。
                           
                           　　进一步强化机遇意识。始终坚持科学发展第一要务不动摇，始终坚持经济建设中心不动摇，善于抢抓转型发展机遇，千方百计用好用活政策、千方百计多上快上项目、千方百计鼓励和引导全民创业、千方百计培植新的经济增长点。
                           
                           　　进一步强化学习意识。坚持笃学慎思、学以致用，不断提高推动发展和驾驭复杂局面、处理复杂问题的本领，提高做好群众工作、维护社会稳定的本领。把改革创新的精神、对外开放的意识融入到政府工作的各个方面，贯穿到政府工作的全过程。
                           
                           　　进一步强化责任意识。责任重于泰山。牢固树立正确的政绩观，坚持以人为本、执政为民。始终牢记党和人民的重托，敢于负责、敢破难题，勇于开拓。倾听群众心声，问政于民，把群众关注的热点、难点问题作为政府工作的出发点和落脚点，真正想群众之所想、急群众之所急、解群众之所难。
                           
                           　　进一步强化实干意识。大兴务实之风，从细节做起，从关键环节着手，察实情、讲实话、创实绩，确保政令畅通。进一步完善责任追究和首办负责、服务承诺等行政效能监察制度，增强政府的执行力和公信力。大兴艰苦奋斗之风，继续压缩政府部门经常性项目支出，严格控制会议、差旅、出国、接待等费用。
                           
                           　　进一步强化法治意识。加强法治型政府建设，提高政府各级领导干部、公务员依法行政意识和能力，努力提供良好的公共服务环境，切实维护社会公平正义。严格执行人大及其常委会的决议、决定，自觉接受人大的法律监督和政协的民主监督，主动接受群众与舆论监督，认真办理人大代表议案、建议和政协委员提案。加强政府立法，提高立法质量；健全执法体制，规范执法行为；强化行政问责，坚决纠正行政不作为和乱作为。
                           
                           　　进一步强化清廉意识。建立健全教育、制度、监督并重的惩治和预防腐败体系，推进政府廉政建设。进一步深化行政审批制度改革，强化行政服务体系建设，不断提高行政效能；加强政务公开，促进行政权力依法公开，阳光运行。进一步完善财政审计监督，推进审计结果公告。加强机关行为规范教育，不断提高政府工作人员素质，努力建设一支忠于职守、清正廉洁、作风优良的公务员队伍。
                           
                           　　各位代表！站在历史的新起点，面对全市人民的新期待，我们深感责任重大、使命光荣。让我们更加紧密地团结在以胡锦涛同志为总书记的党中央周围，深入贯彻落实科学发展观，在省委、省政府和市委的坚强领导下，紧紧依靠和团结全市人民，齐心协力，奋发图强，为全面完成“十二五”规划的各项任务，为把合肥早日建设成区域性特大城市而努力奋斗！
